# Alternative pathway–mediated tubular complement activation in human proteinuric kidney diseases: a proof-of-concept study

**DOI:** 10.1093/ckj/sfaf320

**Published:** 2025-10-16

**Authors:** Firas F Alkaff, Rosa G M Lammerts, Gesa Tiller, Marius C van den Heuvel, Ingeborg M Bajema, Mohamed R Daha, Jacob van den Born, Stefan P Berger

**Affiliations:** Division of Nephrology, Department of Internal Medicine, University of Groningen, University Medical Center Groningen, Groningen, The Netherlands; Division of Pharmacology and Therapy, Department of Anatomy, Histology, and Pharmacology, Faculty of Medicine Universitas Airlangga, Surabaya, Indonesia; Transplantation Immunology, Department of Laboratory Medicine, University of Groningen, University Medical Center Groningen, Groningen, The Netherlands; Division of Nephrology, Department of Internal Medicine, University of Groningen, University Medical Center Groningen, Groningen, The Netherlands; Department of Pathology and Medical Biology, Pathology Section, University of Groningen, University Medical Center Groningen, Groningen, The Netherlands; Department of Pathology and Medical Biology, Pathology Section, University of Groningen, University Medical Center Groningen, Groningen, The Netherlands; Division of Nephrology, Department of Internal Medicine, University of Groningen, University Medical Center Groningen, Groningen, The Netherlands; Department of Nephrology, Leiden University Medical Center, Leiden, The Netherlands; Division of Nephrology, Department of Internal Medicine, University of Groningen, University Medical Center Groningen, Groningen, The Netherlands; Division of Nephrology, Department of Internal Medicine, University of Groningen, University Medical Center Groningen, Groningen, The Netherlands

**Keywords:** chronic kidney disease, complement, proteinuria, proximal kidney tubules, syndecan-1

## Abstract

**Background:**

Proteinuria, irrespective of the primary disease, may cause progressive kidney injury by several mechanisms, including tubular complement activation. Experimental studies have shown that complement activation at the apical side of the tubules is via the alternative pathway, initiated by properdin binding to syndecan-1. However, it has not been systematically studied in human proteinuric kidney diseases.

**Methods:**

Twenty-one kidney biopsies of different proteinuric kidney diseases were stained for complement components MASP-2 (lectin pathway), C1q (classical pathway), properdin (alternative pathway) and downstream complement activation (C3d and C5b-9), as well as syndecan-1. Complement deposition was scored semi-quantitatively. Plasma and urinary soluble C5b-9 were measured using enzyme-linked immunosorbent assays.

**Results:**

All biopsies (21/21) showed apical proximal tubular C3d deposition, of which the majority showed an overlapping distribution with properdin (17/19) and to a lesser extent with C1q (10/19) or C5b-9 (14/21). MASP-2 barely overlapped with the other complement components. Only the intensity and percentage area of the tubular properdin deposition were correlated with that of C5b-9 (ρ = 0.50, *P* = .030 and ρ = 0.50, *P* = .029, respectively). Furthermore, properdin-positive tubules were highly positive for syndecan-1. Proteinuria levels were higher in patients with tubular C5b-9 deposition [7.21 (4.35–11.3) g/10 mmol vs 1.64 (0.56–5.29) g/10 mmol, *P* = .021] and correlated with tubular C5b-9-positive area (ρ = 0.50, *P* = .020). Similarly, patients with detectable urinary soluble C5b-9 also had higher proteinuria levels (*P* = .010).

**Conclusion:**

Apical tubular complement activation in proteinuric patients is mainly via the alternative pathway, potentially initiated by properdin binding to syndecan-1. Inhibition of alternative pathway tubular complement activation may therefore be beneficial for patients with proteinuria.

## Graphical Abstract

**Graphical Abstract:**
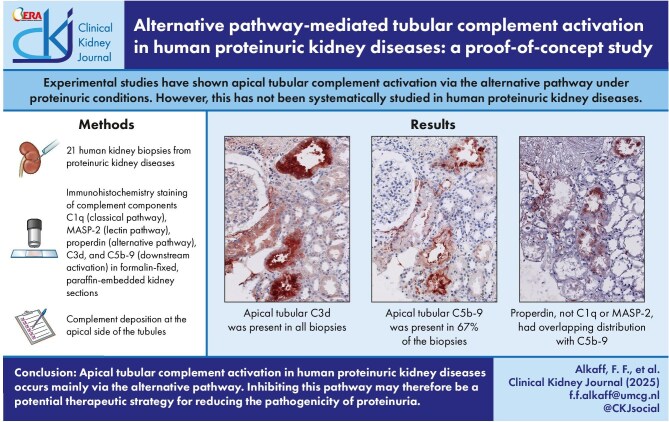

KEY LEARNING POINTS **What was known:**

- Local complement activation is thought to play a pivotal role in proteinuria-induced tubulointerstitial injury.
- Experimental studies have shown that the complement activation at the tubules is via the alternative pathway, initiated by properdin binding to syndecan-1; however, it has not been systematically studied in humans.

**This study adds:**

- C5b-9 deposition in the glomerular area is not correlated with C5b-9 deposition at the apical side of the tubules, indicating that the presence of C5b-9 in the tubules is due to local complement activation rather than spill-over from the circulation.
- Patients with the presence of C5b-9, either in the biopsy or in urine, had higher proteinuria levels.
- Properdin but not C1q or MASP-2 consistently showed overlapping distribution with C5b-9 deposition at the apical side of the tubules, suggesting that the activation pathway is mediated mainly via the alternative pathway.

**Potential impact:**

- Inhibiting the alternative pathway complement activation may be a potential therapeutic strategy for reducing the pathogenicity of proteinuria, regardless of the underlying disease.

## INTRODUCTION

Chronic kidney disease (CKD), defined as abnormalities of kidney structure or function for a minimum of 3 months, is an important contributor to morbidity and mortality from non-communicable disease [1–3]. CKD is a progressive disease, and patients with more advanced CKD have a higher risk of death

compared with those with less advanced CKD. Therefore, the primary goal of CKD management is to slow down CKD progression while striving to achieve the best quality of life and survival [4].

There are many diseases that cause CKD, including hypertension, diabetes and glomerulonephritis [5]. However, irrespective of the underlying etiologies, the progression toward end-stage kidney disease is via one common pathway, i.e. tubulointerstitial injury. An important progression factor that leads to kidney failure via tubulointerstitial injury is proteinuria [6]. Proteinuria induces tubulointerstitial injury through several different mechanisms, including tubular complement activation [6, 7]. Previous studies have shown that the terminal complement complex C5b-9 mediates progressive tubulointerstitial injury in proteinuric animal models but not in non-proteinuric models [8–10]. Similarly, observational studies in patients with different kidney diseases have shown that soluble C5b-9 (sC5b-9) in the urine is associated with tubular injury [11–13].

The complement system can be activated via three different pathways: the classical pathway with C1q as the pattern recognition molecule (PRM) [14]; the lectin pathway with several PRMs [mannose-binding lectin (MBL), ficolins and collectins] that form a complex with MBL-associated serine proteases (MASPs) [15]; and the alternative pathway by spontaneous hydrolysis of C3 or with properdin as the PRM [16]. Regardless of the activation pathway, complement activation will lead to the cleavage of C3 and subsequently result in the formation of C5b-9 [17]. Previous experimental studies have shown that *in vitro* tubular complement activation is via the alternative pathway, with syndecan-1 as the docking platform for the complement activation [18–22]. In line with this, it has been shown that urinary properdin is associated with urinary sC5b-9 and also with worse kidney function, regardless of the underlying kidney diseases [23, 24].

Although there has been a longstanding focus on glomerular complement deposition in kidney research, limited research has been done to systematically evaluate tubular complement deposition in kidney biopsies from patients with proteinuric kidney diseases [25, 26]. Therefore, this study aims to evaluate the complement component deposition of each activation pathway (C1q for classical pathway, MASP-2 for lectin pathway and properdin for alternative pathway), the downstream complement activation (C3d and C5b-9), and also the properdin docking receptor syndecan-1 at the apical side of the tubules in patients with a number of different proteinuric kidney diseases. We hypothesized that C5b-9 is deposited at the apical side of the tubules in patients with proteinuric kidney disease, irrespective of the underlying cause, and that the amount of the deposition is correlated with the degree of proteinuria. Furthermore, we also hypothesized that when C5b-9 is present, the deposition pattern overlaps with properdin and not with C1q or C4d, similar to what has been shown by the experimental studies [18–22].

## MATERIALS AND METHODS

This study adhered to the Declaration of Helsinki and was approved by the University Medical Center Groningen (UMCG) institutional review board (Research registry number 202 000 076, approved on 29 March 2021). Patients gave written informed consent for the use of their kidney biopsy material and access to their medical records.

This cross-sectional study included patients whose blood samples had been taken for direct complement measurement and who had undergone an indication kidney biopsy at the UMCG, The Netherlands, between 2012 and 2014 as a part of the standard patient care. In some patients, urine samples were also collected at the same time as the blood samples. The blood and urine samples were immediately put on ice and stored at –80°C freezer, and only thawed directly before complement measurement to prevent *in vitro* complement activation. Post-transplant patients, patients with normal kidney biopsy findings, patients without leftover formalin-fixed paraffin-embedded kidney biopsies for additional immunohistochemistry (IHC) staining and patients aged below 16 years old when informed consent was requested were excluded. From the inclusion and exclusion criteria, kidney biopsies from 21 patients with different primary glomerular kidney diseases [3 patients with lupus nephritis, 3 patients with focal segmental glomerulosclerosis, 6 patients with IgA nephropathy (IgAN), 2 patients with diabetic nephropathy, 2 patients with mesangioproliferative glomerulonephritis, 3 patients with membranous nephropathy and 2 patients with minimal change disease] were used. To confirm whether complement activation may also occur in a condition where there was no glomerular injury, we additionally included biopsies from three patients with tubulointerstitial nephritis (TIN) without any glomerular abnormalities. The biopsies were cut consecutively at 3 µm thickness and stained for syndecan-1 and complement components C5b-9, C3d, properdin, C1q and MASP-2. When two complement stainings were observed in the same tubular regions across serial sections, this was described as having an overlapping distribution. A deceased human kidney donor not suitable for transplantation for anatomical reasons was used for the positive control. For the negative control, the primary antibody in the kidney donor was replaced with phosphate-buffered saline. The detailed staining protocol is presented in Supplementary data, Table S1, and the representative staining of the control sample is presented in Supplementary data, Fig. S1

Kidney biopsies were reviewed for complement deposition intensity (–, ±, +, ++ and +++) at the glomeruli and apical side of the tubules and also for the percentage of deposition area at the apical side of the tubules (0%, 1%–10%, 11%–25%, >25% and >50%) by two observers (F.F.A. and G.T.) who were blinded to the clinical data. All disagreements were resolved by the senior researcher (J.v.d.B.) who was also blinded to the clinical data. Relevant clinical data were collected from the electronic medical record. Creatinine was measured using an enzymatic assay that is traceable to isotope dilution mass spectroscopy by Roche, whereas protein concentration was measured using a colorimetric assay by Roche [27]. Estimated glomerular filtration rate was calculated using the creatinine-based Chronic Kidney Disease Epidemiology Collaboration equation for patients ≥18 years old and the creatinine-based ‘Bedside Schwartz’ equation for patients aged <18 years old [28, 29]. Proteinuria was expressed as g/10 mmol creatinine.

Plasma and urinary sC5b-9 levels were assessed in duplo by a previously standardized and validated sandwich enzyme-linked immunosorbent assay (ELISA) with a limit of detection (LOD) of 8.6 ng/mL. The sC5b-9 ELISA protocol has been described in detail elsewhere [24, 30]. For urinary sC5b-9, it was presented both in its concentration and corrected for urinary creatinine level. When the sC5b-9 concentration was below the LOD, a value of 4.3 ng/mL (half of the LOD) was given.

Continuous data were presented as median (interquartile range), and nominal data were presented as frequency (valid percentage). The Mann–Whitney U test was used to compare the differences of continuous variables between the two groups. Spearman rank correlation was used to evaluate the correlation between ordinal and ordinal or between ordinal and continuous variables. All data were analyzed using SPSS version 25.0 (IBM Corp., Armonk, NY, USA). For all analyses, *P*-value <.05 was considered statistically significant.

## RESULTS

The individual patients’ clinical data with glomerular kidney disease are presented in Table 1. Two patients (9.5%) underwent biopsy because of kidney function decline, 10 patients (47.6%) because of proteinuria, and 9 patients (42.9%) because of both kidney function decline and proteinuria. None of the patients was on dialysis treatment nor underwent kidney transplantation.

**Table 1:** Clinical characteristics of each patient in the study population.

Patients number	Diagnosis	Age (years)	Sex	Serum creatinine (µmol/L)	eGFR (mL/min/1.73 m^2^)	PCR (g/10 mmol)	Anti-proteinuric medication
1	LN	31	Male	90	100.99	7.67	Yes
2	LN	42	Male	198	36.62	5.14	No
3	LN	49	Male	73	107.45	1.64	Yes
4	MN	26	Male	77	122.00	6.92	Yes
5	MN	42	Male	260	26.41	10.57	Yes
6	MN	55	Female	169	30.57	5.61	Yes
7	MPGN	8	Female	40	130.32	1.90	Yes
8	MPGN	8	Female	54	88.57	13.57	No
9	IgAN	28	Male	79	119.56	8.95	Yes
10	IgAN	72	Male	179	34.29	2.18	Yes
11	IgAN	24	Female	66	114.57	2.00	Yes
12	IgAN	37	Male	131	62.01	0.64	No
13	IgAN	57	Male	127	56.83	1.63	No
14	IgAN	75	Male	210	27.79	0.56	Yes
15	DN	41	Female	204	26.61	5.29	Yes
16	DN	68	Female	144	34.17	0.15	Yes
17	FSGS	53	Male	160	44.16	5.28	Yes
18	FSGS	51	Male	223	30.02	9.30	Yes
19	FSGS	66	Male	334	16.84	13.68	Yes
20	MCD	69	Male	137	48.16	7.50	Yes
21	MCD	16	Male	65	99.98	14.17	Yes

The use of anti-proteinuric medication was defined as the use of either an angiotensin-converting enzyme inhibitor, angiotensin receptor blocker, or diuretics at the time of biopsy.

IgAN, immunoglobulin A nephropathy; DN, diabetic nephropathy; eGFR, estimated glomerular filtration rate based on creatinine-based Chronic Kidney Disease Epidemiology Collaboration equation for patients ≥18 years old and creatinine-based ‘Bedside Schwartz’ equation for patients aged <18 years old; FSGS, focal segmental glomerulosclerosis; LN, lupus nephritis; MCD, minimal change disease; MN, membranous nephropathy; MPGN, membranoproliferative glomerulonephritis; PCR, protein/creatinine ratio.

C3d deposition at the apical side of the tubules was present in all (21/21) biopsies, albeit the deposition in the glomeruli was only detected in 75% of the patients. No correlation was seen between the C3d deposition intensity at the glomeruli and the apical side of the tubules (Spearman ρ = –0.03, *P* = .9).

C5b-9 deposition at the apical side of the tubules was observed in 67% (14/21) of the biopsies, whereas C5b-9 deposition at the glomeruli was observed in 60% (12/20) of the biopsies. There was no correlation between the C5b-9 deposition intensity at the apical side of the tubules and at the glomeruli (Spearman ρ = 0.21, *P* = .4). Of these 14 biopsies that were positive for C5b-9 at the apical side of the tubules, C3d deposition showed an overlapping distribution with C5b-9 in 13 of them (93%). The intensity of the tubular C5b-9 deposition was not significantly correlated with that of C3d (Spearman ρ = 0.42, *P* = .062). Patients with C5b-9 deposition at the apical side of the tubules had similar kidney function levels compared with patients without C5b-9 deposition; however, proteinuria levels were significantly higher in patients with C5b-9 deposition [7.21 (4.35–11.3) g/10 mmol vs 1.64 (0.56–5.29) g/10 mmol, *P* = .021] (Table 2). Furthermore, proteinuria levels significantly correlated with the percentage area of the C5b-9 deposition at the apical side of the tubules (Spearman ρ = 0.50, *P* = .020).

**Table 2:** Clinical characteristics stratified based on the presence of C5b-9 deposition at the apical side of the tubules.

Variables	Absent, *N* = 7	Present, *N* = 14	*P*-value
Age, years	57 (41–72)	40 (22–54)	.052
Female sex	2 (29)	4 (29)	1.0
Serum creatinine, µmol/L	144 (90–204)	134 (66–204)	.6
eGFR, mL/min/1.73 m^2^	34.3 (27.8–101)	55.1 (30.4–116)	.5
PCR, g/10 mmol	1.64 (0.56–5.29)	7.21 (4.35–11.3)	.021
Use of anti-proteinuric medication	6 (86)	11 (79)	1.0

Data are presented as median (interquartile range) or *n* (%).

The use of anti-proteinuric medication was defined as the use of either an angiotensin-converting enzyme inhibitor, angiotensin receptor blocker, or diuretics at the time of biopsy.

eGFR, estimated glomerular filtration rate based on creatinine-based Chronic Kidney Disease Epidemiology Collaboration equation for patients ≥18 years old and creatinine-based ‘Bedside Schwartz’ equation for patients aged <18 years old; PCR, protein/creatinine ratio.

There were 19 biopsies stained for properdin. Both properdin deposition at the apical side of the tubules and at the glomeruli were observed in 89% (17/19) of the biopsies. There was a significant correlation between properdin deposition intensity at the apical side of the tubules and at the glomeruli (Spearman ρ = 0.65, *P* = .002). Within the C5b-9-positive biopsies (*N* = 14), 13 were stained for properdin as well, and distribution between properdin and C5b-9 can be assessed in 12 of them. All (12/12) had an overlapping distribution with properdin at the apical side of the tubules. Furthermore, the intensity and the percentage area of the tubular properdin deposition were correlated with that of C5b-9 (Spearman ρ = 0.50, *P* = .030 and Spearman ρ = 0.50, *P* = .029, respectively).

There were 18 biopsies stained for C1q. C1q deposition at the apical side of the tubules and at the glomeruli was observed in 55.6% (10/18) and 72% (13/18) of the biopsies, respectively. There was a significant correlation between C1q deposition intensity at the apical side of the tubules and the glomeruli (Spearman ρ = 0.49, *P* = .039). Apical tubular C1q distribution (10/18) largely overlapped with properdin (8/15) and C3d (8/17), and to a lesser extent with C5b-9 (5/17). The intensity and the percentage area of the tubular C1q deposition were not correlated with the other complement stainings.

There were 17 biopsies stained for MASP-2. Twelve out of 17 were positive for MASP-2 at the apical side of tubules, whereas MASP-2 deposition at the glomeruli was absent in all patients. However, unlike other complement components, MASP-2 was deposited mainly at the distal tubules (based on morphological appearance) instead of the proximal tubules, and the distribution barely overlapped with the other complement stainings. The intensity and the percentage area of the tubular MASP-2 deposition were not correlated with that of the other complement stainings.

**Figure 1:**
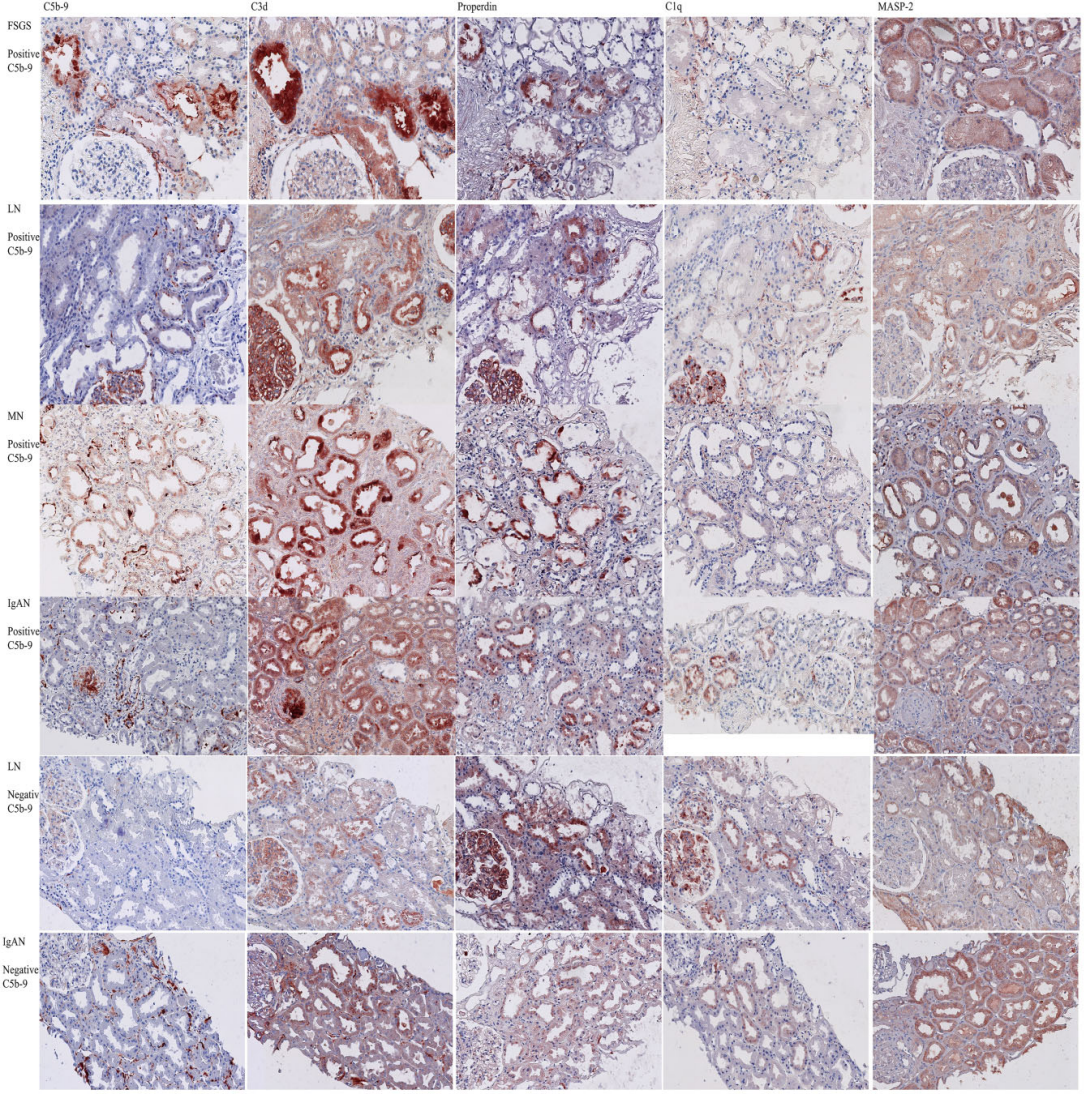
The staining of complement components in kidney biopsies of patients with positive and negative C5b-9 deposition at the apical side of the tubules.

**Figure 2:**
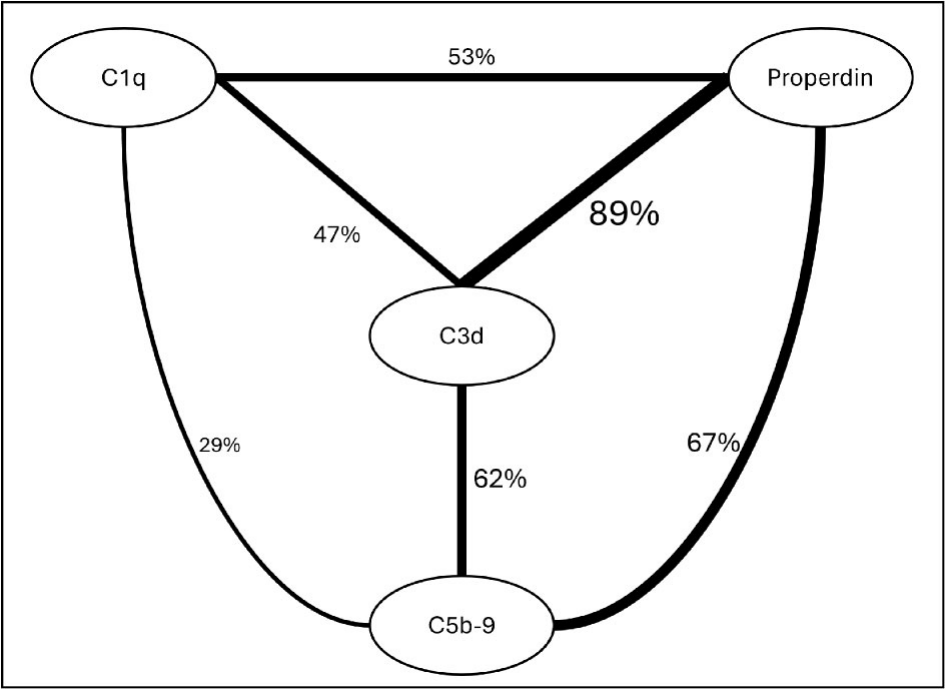
Summary of the complement components’ positivity and their overlapping distribution with other complement components. MASP-2 was not included as the deposition barely overlapped with other complement components.

The representatives of complement components staining in the kidney biopsies are presented in Fig. 1. The detailed complement deposition score at the apical side of the tubules of each patient is presented in Supplementary data, Table S2 The positivity for complement components and their overlapping distribution with other complement components is summarized in Fig. 2.

**Figure 3:**
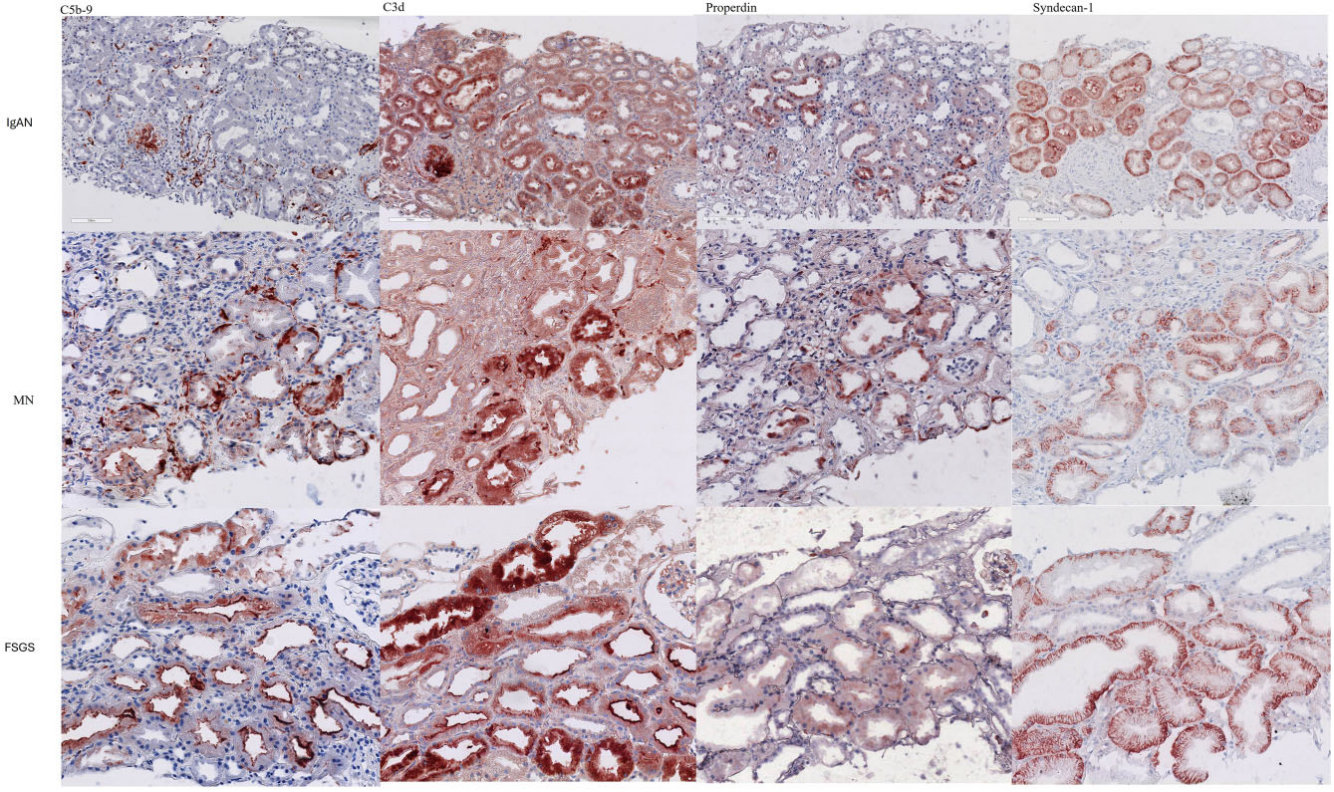
C5b-9, C3d, properdin and syndecan-1 staining in human kidney biopsies. Syndecan-1 is expressed in the tubular basement membrane of proximal tubules but not in distal tubules. When properdin is deposited, syndecan-1 is also expressed at the apical side of the tubules. FSGS, focal segmental glomerular sclerosis; MN, membranous nephropathy.

In all biopsies, expression of syndecan-1 was observed in the basolateral cell membranes of PTEC. In a number of biopsies, however, the lateral syndecan-1 staining reached the apical cell membranes, and sometimes, the whole apical tubular cell membranes were positive for syndecan-1 as well. Importantly, when there was properdin deposition at the apical side of the tubules, syndecan-1 was also expressed at the same location, either via the extended lateral or via a complete apical expression pattern (Fig. 3).

Of 21 patients, 14 had blood and urine samples collected at the time of biopsy. Median plasma sC5b-9 levels were 34.5 (31–84) ng/mL. For urinary sC5b-9, six (43%) patients had levels below the LOD. There was no correlation between plasma sC5b-9 and either urinary sC5b-9 concentration or urinary sC5b-9/creatinine ratio (ρ = –0.37, *P*-value = .2 and ρ = –0.48, *P*-value = .084, respectively). Similar to findings from the C5b-9 deposition biopsy, patients with detectable urinary sC5b-9 also had higher proteinuria levels [7.28 (2.96–10.25) ng/mL vs 1.15 (0.46–2.81) ng/mL, *P* = .010]. However, three of eight patients with C5b-9 deposition at the apical side of the tubules had undetectable urinary sC5b-9. Conversely, three of six patients without C5b-9 deposition at the apical side of the tubules had detectable urinary sC5b-9 (Supplementary data, Table S3).

Furthermore, similar to the biopsy findings where the percentage area of the C5b-9 deposition at the apical side of the tubules were correlated with proteinuria levels, urinary sC5b-9 concentration and urinary sC5b-9/creatinine ratio were also correlated with the proteinuria level (ρ = 0.85, *P*-value <.001, and ρ = 0.78, *P*-value = .001, respectively), whereas plasma sC5b-9 was not (ρ = 0.10, *P*-value = .7). Nonetheless, there was no correlation between the percentage area of the C5b-9 deposition at the apical side of the tubules with the urinary sC5b-9 concentration or urinary sC5b-9/creatinine ratio (both ρ = 0.30, *P*-value = .3).

We additionally included three patients with TIN, to explore whether tubular complement activation also occurs in patients without glomerular injury. The clinical characteristics of these patients are presented in Supplementary data, Table S4 None of them had C5b-9 deposition at the apical side of the tubules. Furthermore, urinary sC5b-9 level was also undetectable in these patients (Supplementary data, Table S5). The C5b-9 stainings of the TIN patients are presented in Supplementary data, Fig. S2.

## DISCUSSION

This study is to our knowledge the first to systematically evaluate complement activation in human kidney tissue at the apical side of the tubules in various proteinuric kidney diseases. This study showed that, irrespective of the underlying kidney diseases, proteinuria was associated with local complement activation at the apical side of the tubules, as evidenced by C5b-9 positivity. The majority (89%) of the apical tubular C3d depositions showed an overlapping distribution with properdin, and to a lesser extent (62%) also with C5b-9, in 47% with C1q, and not at all with MASP-2, indicating predominant alternative tubular complement activation under proteinuric conditions. Next to that, syndecan-1, reported to function as a properdin docking structure, was expressed at the lateral/apical side of the tubules under proteinuric conditions, and its distribution overlapped with properdin. These results indicate that aberrantly filtrated properdin might initiate proximal tubular alternative complement activation not only in tubular cell culture systems and rodent proteinuria models but also in human proteinuric kidney diseases.

Under healthy conditions, only low molecular weight proteins (below 40 kDa) are completely unrestricted to pass the glomerular filtration barrier (GFB) and enter the tubular lumen, whereas only a fraction of intermediate molecular weight proteins (particularly albumin with a molecular weight of 69 kDa) and none of the high molecular weight protein is able to pass [31]. Complement components have different molecular weights ranging from 23 to 400 kDa, and the majority have high molecular weights [32]. C5b-9, since it is a complex, has an even larger molecular weight, i.e. 1000 kDa [33]. Therefore, tubular complement activation does not occur in non-proteinuric conditions [10].

When the glomeruli are injured, the GFB is disrupted. This will lead to an increasing amount of larger molecular weight protein that is able to pass through the GFB and enter the tubular lumen. In the less severe cases, only intermediate molecular weight proteins enter the tubular lumen, and this condition is called selective proteinuria. When the glomerular injury becomes more severe, heavy molecular weight proteins are also present in the tubular lumen, and this condition is called non-selective proteinuria [31]. Because most of the tubular complement components have a large molecular weight, tubular complement activation only occurs during non-selective proteinuria, as shown by previous experimental studies [8, 9, 34]. In line with this, we observed no tubular complement activation in patients with TIN without glomerular injury, as reflected by negative C5b-9 deposition in the biopsy or undetectable sC5b-9 level in the urine.

However, although heavy molecular weight proteins can pass through the GFB during non-selective proteinuria, that is not the case for C5b-9. A previous study has shown that even though the molecular weight of C5b-9 is similar to that of IgM, the fractional excretion of C5b-9 is 100 times higher than that of IgM [35]. Previous observational studies have also shown that there is no correlation between plasma and urinary sC5b-9 levels, regardless of the type of kidney disease [13, 36–39]. In line with this, our study also showed that there is no correlation between C5b-9 deposition in the glomeruli and the apical side of the tubules, or between plasma and urinary sC5b-9 levels. This indicates that the presence of C5b-9 deposition at the apical side of the tubules is due to intratubular complement activation and not because of the spillover from the circulation. In addition, we found that patients with C5b-9 deposition at the apical side of the tubules had significantly higher proteinuria, and that the level of proteinuria was positively correlated with the affected area of the C5b-9 deposition. Similarly, we also found that urinary sC5b-9 level was significantly correlated with proteinuria level. This is in line with what has been shown by the earlier studies [36, 37, 40].

We found that some patients with C5b-9 deposition at the apical side of the tubules had undetectable urinary sC5b-9. To our knowledge, only one previous study has assessed the C5b-9 deposition in the tubules and urinary sC5b-9 level simultaneously [36]. That study also found that C5b-9 can be present at the apical side of the tubules, even when the urinary sC5b-9 level was low [36]. One possible explanation is that this may be a result of focal disease and thus localized deposition in a smaller number of affected nephrons but insufficient tubular complement activation to increase urine C5b-9 levels. When most of the kidney is unaffected, the overall urinary sC5b-9 concentration is likely to remain low.

Conversely, we also found that some patients without C5b-9 deposition at the apical side of the tubules had detectable urinary sC5b-9, including one case with a markedly high concentration (344 ng/mL). This discrepancy might be attributable to sampling limitations inherent to kidney biopsy, which represents only a small fraction of the kidney. Because complement deposition can be segmental, the absence of C5b-9 in the biopsy specimens does not necessarily exclude its presence elsewhere. Future studies that concurrently evaluate C5b-9 deposition at the apical side of the tubules and urinary sC5b-9 levels are warranted to validate these findings.

We found that C3d was present at the apical side of the tubules in all biopsies, which were also positive for C5b-9. Additionally, most of the C3d deposition showed an overlapping distribution with C5b-9 deposition. However, in contrast to C5b-9, there was no correlation between C3d deposition and proteinuria. Furthermore, neither the intensity nor the percentage area of the tubular C3d deposition was correlated with tubular C5b-9 deposition. While the only source of C5b-9 is tubular complement activation, C3 may have originated from the circulation or local production by glomerular or tubular cells [41]. Previously, it has been reported that ultrafiltered C3 but not local C3 synthesis contributes to the development of progressive renal injury [42]. However, our staining cannot distinguish between these two sources of C3d. This might potentially explain the difference in the association of C3d or C5b-9 with the degree of proteinuria and also the absence of the correlation between tubular C3d and C5b-9 deposition.

We found that properdin was deposited at the apical side of the tubules in the same location as C3d and C5b-9 deposition, and the intensity and the percentage area of the tubular properdin deposition were significantly correlated with tubular C5b-9 deposition. While previous studies showed intratubular alternative pathway complement activation in the experimental setting and also in the urine of the patients [18–24], the current study shows that this is indeed the case at the human tissue level. While previous experimental studies using PTEC cultures showed that C1q did not play a role in tubular complement activation [19, 20, 43], we found that C1q was deposited at the apical side of the tubules in around half of the biopsies, and the majority of C1q showed an overlapping distribution with properdin, C3d and C5b-9. However, whether C1q deposition contributes to tubular complement activation by initiating the classical pathway or is merely a bystander because it passes through the filtration barrier and is reabsorbed by the PTEC remains to be elucidated. MASP-2, on the other hand, was deposited mainly at the distal tubules. Since none of the other complement factors was also deposited at the distal tubules, this suggests that the lectin pathway is not involved in the tubular complement activation.

We found that basal expression of syndecan-1 was in the basolateral cell membranes of the proximal tubular epithelial cells, similar to what has been shown previously [44, 45]. Next, we observed that when properdin was deposited at the apical side of the tubules, syndecan-1 was expressed in an extended lateral and/or apical fashion in addition to the basolateral cell membranes. This supports the notion from previous studies that syndecan-1 is a docking platform for properdin to initiate the complement activation at the apical side of the tubules [21, 22].

Because of the detrimental effects of proteinuria on the progression of CKD toward end-stage kidney disease, there is a need to reduce proteinuria as much as possible. However, with the current medications, such as renin–angiotensin–aldosterone inhibitors or sodium-glucose co-transporter 2 inhibitors [46, 47], proteinuria is only partially suppressed. Considering that tubular complement activation is one of the main drivers of proteinuria-induced CKD progression [7], and that a large body of evidence (including our current study) indicates that tubular complement activation occurs via the alternative pathway, combining anti-proteinuric medication with complement inhibitors that target the alternative pathway might be a potential therapeutic approach. Currently, there is one drug that inhibits the alternative pathway in the last phases of clinical trials for kidney disease, namely iptacopan (a factor B inhibitor) [48]. In a recent phase 3 clinical trial, ipcatopan has been shown to significantly reduce the urinary sC5b-9 level in patients with IgAN. Interestingly, this effect on urinary sC5b-9 is far more marked than the proteinuria reduction [49]. Similar findings were also reported in the phase 2 clinical trial for patients with C3 glomerulopathy [50]. This suggests that the marked reduction of sC5b-9 excretion is largely caused by the inhibition of tubular complement activation rather than by the reduction of proteinuria. Considering that tubular complement activation contributes to tubulointerstitial injury regardless of the underlying cause, inhibiting the alternative pathway complement activation might be a future therapeutic approach for preventing progressive kidney function loss in patients with any chronic non-selective proteinuric kidney diseases. Nonetheless, for complement inhibition to be clinically meaningful, it should lead to improvement in clinical parameters, particularly kidney function. This remains to be seen at the end of the 2-year double blind iptacopan treatment [49].

The important limitations in the present study are that we only included biopsies from 21 patients, and the blood and urine samples were only available in 14 of them. Nevertheless, this is the first study to evaluate the complement component deposition of all potential activation pathways and the downstream complement activation at the apical side of the tubules at the human tissue level.

## CONCLUSION

This study is the first to show on human kidney tissue that C5b-9 is deposited on the tubular brush border in various proteinuric human kidney diseases, with the activation mediated mainly via the alternative pathway, potentially initiated by properdin binding to syndecan-1. Inhibiting the alternative pathway complement activation may therefore be a potential therapeutic strategy for reducing the pathogenicity of proteinuria, regardless of the primary disease.

## Supplementary Material

sfaf320_Supplemental_File

## Data Availability

The data supporting this study’s findings are not publicly available due to privacy policies and data protection regulations, but they are available from the corresponding author upon reasonable request.
