# Supplementary material for: Alternative pathway–mediated tubular complement activation in human proteinuric kidney diseases: a proof-of-concept study
Source: Clin Kidney J. 2025 Oct 16;18(11):sfaf320. doi: 10.1093/ckj/sfaf320 (PMC12596484; doi:10.1093/ckj/sfaf320)
Supplement: sfaf320_Supplemental_File [file sfaf320_supplemental_file.pdf]

Alternative pathway-mediated tubular complement activation in human proteinuric kidney diseases: A proof-of-concept study

SUPPLEMENTAL MATERIAL

Table of contents

**Supplementary Figure 1.** Complement component and syndecan-1 staining in the control sample.	Page 2
**Supplementary Figure 2.** Complement C5b-9 staining in tubulointerstitial nephritis biopsies.	Page 3
**Supplementary Table 1.** Details on immunohistochemistry in formalin-fixed paraffin-embedded kidney biopsies.	Page 4
**Supplementary Table 2.** Complement components' score at the apical side of the tubules of each patient.	Page 7
**Supplementary Table 3.** Complement C5b-9 score in the biopsy and sC5b-9 level in plasma and urine of each patient.	Page 9
**Supplementary Table 4.** Clinical characteristics of each patient with tubulointerstitial nephritis.	Page 10
**Supplementary Table 5.** Complement components' score at the apical side of the tubules of each patient with tubulointerstitial nephritis and its plasma and urinary sC5b-9 level.	Page 11

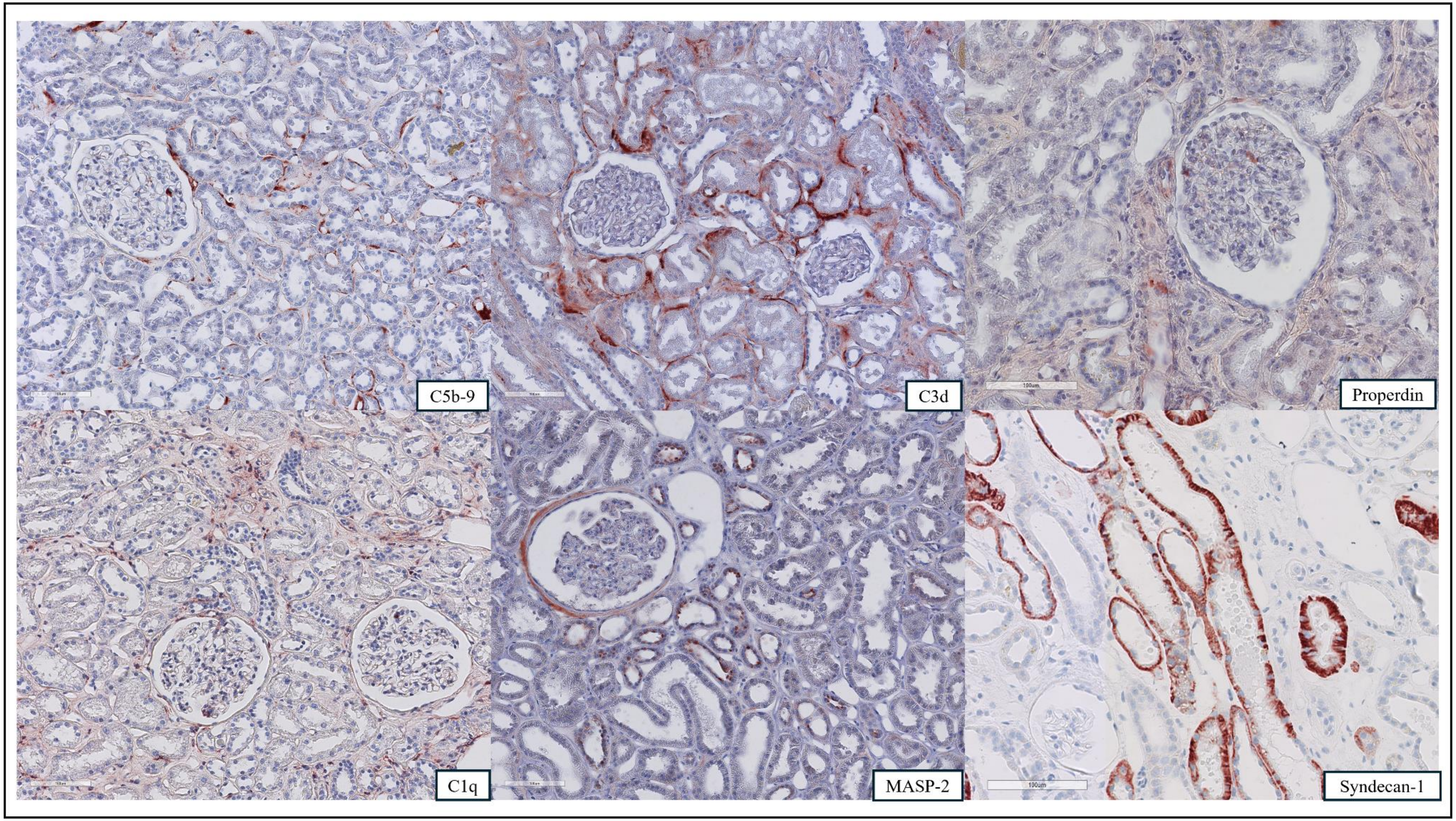

Supplementary Figure 1. Complement component and syndecan-1 staining in the control sample.

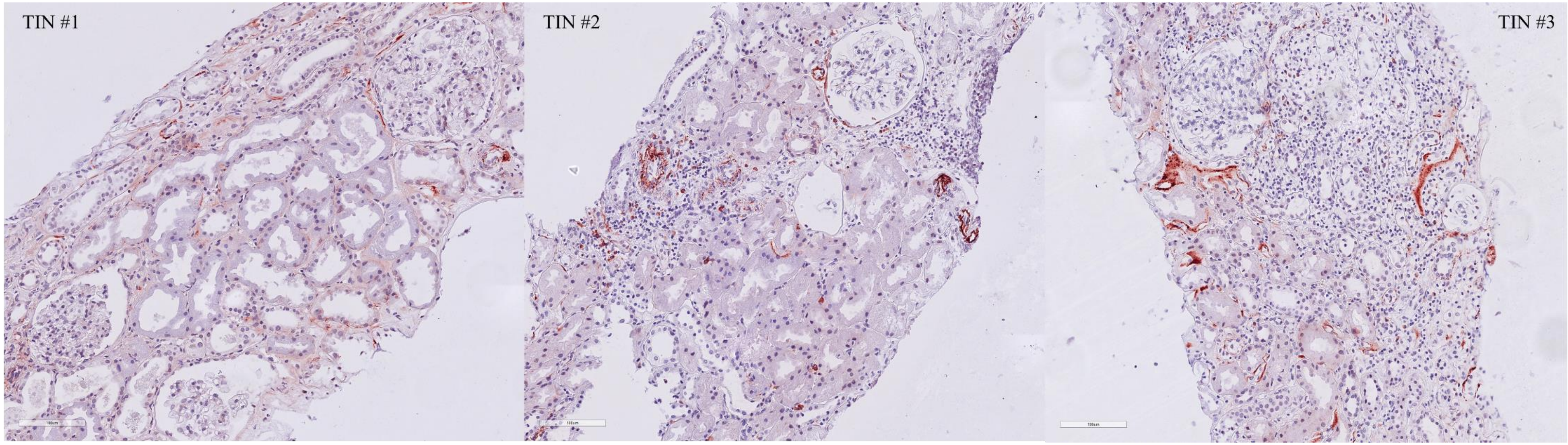

Supplementary Figure 2. Complement C5b-9 staining in tubulointerstitial nephritis biopsies.

Supplementary Table 1. Details on immunohistochemistry in formalin-fixed paraffin-embedded kidney biopsies.

Antigen	Antigen retrieval	Blocking step	Antibody	Conjugate 1	Conjugate 2	Substrate	Control sample
C5b-9	0.1% protease for 30 minutes at room temperature	0.3% H_2O_2 for 30 minutes at room temperature	Monoclonal mouse anti-human (Quidel, A239), 1:10,000 dilution in 1% BSA, overnight at 4ºC	polyclonal rabbit anti-mouse HRP (DAKO, P0260), 1:100 dilution in 1% BSA + 1% NHS, for 30 minutes at room temperature	polyclonal goat anti-rabbit HRP (DAKO, P0448), 1:100 dilution in 1% BSA + 1% NHS for 30 minutes at room temperature	Ready-to-use AEC substrate kit (ABCAM ab64252, undiluted) at room temperature for 20 minutes	Pre-transplantation kidney biopsy of a deceased donor
C3d	0.4% pepsin for 30 minutes at room temperature		polyclonal rabbit anti-human (DAKO, A0063), 1:2,500 dilution in 1% BSA, overnight at 4ºC	polyclonal goat anti-rabbit HRP (DAKO, P0448), 1:100 dilution in 1% BSA, for 30 minutes at room temperature	polyclonal rabbit anti-goat HRP (DAKO, P0449), 1:100 dilution in 1% BSA, for 30 minutes at room temperature		

Properdin	0.1% protease for 30 minutes at room temperature		polyclonal rabbit anti-human (Laboratory of Nephrology, Leiden, the Netherlands), 1:400 dilution in 1% BSA, overnight at 4°C				
C1q	0.1% protease for 30 minutes at room temperature		Polyclonal rabbit anti-human (DAKO, A0136), 1:3,200 dilution in 1% BSA, 60 minutes at room temperature				

MASP-2	Pressure cooker at 125°C for 15 min in Citrate buffer 10 mM pH 6		Polyclonal rabbit anti-human (Sigma-Aldrich, HPA029313), 1:100 dilution in 1% BSA, overnight at 4ºC				
Syndecan-1		0.3% H2O2 for 30 minutes, followed by 5% BSA for 30 minutes at room temperature	Polyclonal rabbit anti-human (BioRad, MCA2459T), 1:400 dilution in 1% BSA, overnight at room temperature	polyclonal goat anti-rabbit HRP (DAKO, P0448), 1:100 dilution in 1% BSA + 1% NHS, for 30 minutes at room temperature	polyclonal rabbit anti-goat HRP (DAKO, P0449), 1:100 dilution in 1% BSA + 1%NHS, for 30 minutes at room temperature		

Supplementary Table 2. Complement components' score at the apical side of the tubules of each patient.

Patients Number	Disease	PCR (g/10mmol)	C5b-9		C3d		Properdin		C1q		MASP-2	
			Intensity	%area	Intensity	%area	Intensity	%area	Intensity	%area	Intensity	%area
**C5b-9-positive**												
2	LN	5.14	+++	1-10%	+++	26-50%	+++	11-25%	++	1-10%	-	0%
4	MN	6.92	+++	>50%	+++	>50%	++	11-25%	+/-	1-10%	++	1-10%
5	MN	10.57	+++	11-25%	+++	11-25%	+++	11-25%	+	1-10%	++	1-10%
6	MN	5.61	+++	1-10%	++	1-10%	+++	1-10%	+	1-10%	-	0%
7	MPGN	1.90	+	1-10%	+	1-10%	+	1-10%	[redacted]	[redacted]	-	0%
8	MPGN	13.57	+	1-10%	++	26-50%	+	11-25%	+++	26-50%	-	0%
9	IgAN	8.95	+	1-10%	++	11-25%	+/-	>50%	[redacted]	[redacted]	[redacted]	[redacted]
11	IgAN	2.00	++	1-10%	+++	26-50%	++	11-25%	++	11-25%	+	11-25%
12	IgAN	0.64	+++	1-10%	+	11-25%	[redacted]	[redacted]	-	0%	[redacted]	[redacted]
17	FSGS	5.28	+++	11-25%	+++	26-50%	++	11-25%	-	0%	+++	1-10%
18	FSGS	9.30	+++	11-25%	+++	11-25%	++	11-25%	-	0%	++	1-10%
19	FSGS	13.68	+	1-10%	++	1-10%	+	1-10%	-	0%	++	1-10%
20	MCD	7.50	+/-	1-10%	+	1-10%	+	11-25%	+	11-25%	[redacted]	[redacted]
21	MCD	14.17	+/-	1-10%	+	11-25%	+	1-10%	-	0%	[redacted]	[redacted]
**C5b-9-negative**												

1	LN	7.67	-	0%	+++	>50%	++	1-10%	-	0%	+++	26-50%
3	LN	1.64	-	0%	+	11-25%	+++	26-50%	++	1-10%	++	1-10%
10	IgAN	2.18	-	0%	++	26-50%	++	1-10%	+	1-10%	+	26-50%
13	IgAN	1.63	-	0%	++	11-25%	+	1-10%	++	1-10%	++	1-10%
14	IgAN	0.56	-	0%	+	1-10%			-	0%	++	1-10%
15	DN	5.29	-	0%	+++	>50%	-	0%			-	0%
16	DN	0.15	-	0%	+	26-50%	-	0%	-	0%	++	1-10%

IgAN, immunoglobulin A nephropathy; DN, diabetic nephropathy; FSGS, focal segmental glomerulosclerosis; LN, lupus nephritis; MCD, minimal change disease; MN, membranous nephropathy; MPGN, membranoproliferative glomerulonephritis.

Supplementary Table 3. Complement C5b-9 score in the biopsy and sC5b-9 level in plasma and urine of each patient.

Patients Number	Disease	PCR (g/10mmol)	C5b-9		Plasma C5b-9	Detectable urinary sC5b-9	Urinary sC5b-9 concentration	Urinary sC5b-9/creatinine ratio
			Intensity	%area	ng/ml		ng/ml	ng/mmol
5	MN	10.57	+++	11-25%	30	detectable	550	78.57
6	MN	5.61	+++	1-10%	31	detectable	281	49.30
9	IgAN	8.95	+	1-10%	37	detectable	403	70.70
11	IgAN	2.00	++	1-10%	61	not detectable	4.3	1.23
12	IgAN	0.64	+++	1-10%	178	not detectable	4.3	.34
17	FSGS	5.28	+++	11-25%	37	not detectable	4.3	.60
18	FSGS	9.30	+++	11-25%	36	detectable	28	2.80
19	FSGS	13.68	+	1-10%	47	detectable	550	63.22
3	LN	1.64	-	0%	313	not detectable	4.3	.32
10	IgAN	2.18	-	0%	159	detectable	14	2.55
13	IgAN	1.63	-	0%	31	detectable	23.	2.50
14	IgAN	0.56	-	0%	32	not detectable	4.3	.61
15	DN	5.29	-	0%	24	detectable	344	25.29
16	DN	0.15	-	0%	29	not detectable	4.3	1.59

IgAN, immunoglobulin A nephropathy; DN, diabetic nephropathy; FSGS, focal segmental glomerulosclerosis; LN, lupus nephritis; MCD, minimal change disease; MN, membranous nephropathy; MPGN, membranoproliferative glomerulonephritis; sC5b-9, soluble C5b-9.

Supplementary Table 4. Clinical characteristics of each patient with tubulointerstitial nephritis.

Patient	Age (years)	Sex	Serum creatinine (µmol/L)	eGFR (mL/min/1.73m^2)	PCR (g/10mmol)	Anti-proteinuric medication
1	69	male	179	34.94	.06	no
2	63	female	152	33.03	.10	no
3	80	female	128	36.53	.39	no

eGFR, estimated glomerular filtration rate based on creatinine-based CKD-EPI equation. PCR, protein/creatinine ratio. The use of anti-proteinuric medication was defined as the use of either an angiotensin-converting enzyme inhibitor, angiotensin receptor blocker, or diuretics at the time of biopsy.

Supplementary Table 5. Complement components' score at the apical side of the tubules of each patient with tubulointerstitial nephritis and its plasma and urinary sC5b-9 level.

Patient	PCR (g/10mmol)	C5b-9		C3d		Properdin		C1q		Urinary sC5b-9 level	Plasma sC5b-9 level (ng/ml)
		Intensity	%area	Intensity	%area	Intensity	%area	Intensity	%area		
1	0.06	-	0%	-	0%	-	0%	-	0%	Undetectable	23
2	0.10	-	0%	-	0%	-	0%	-	0%	Undetectable	32
3	0.39	-	0%	-	0%	-	0%	-	0%	Undetectable	37

TIN, tubulointerstitial nephritis; PCR, protein/creatinine ratio; sC5b-9, soluble C5b-9. The lowest limit of detection for the sC5b-9 was 8.6 ng/ml.
